# Giant Paratesticular Liposarcoma Mimicking a Left-Sided Groin Hernia: A Case Report

**DOI:** 10.7759/cureus.28856

**Published:** 2022-09-06

**Authors:** Kimberley Chan, Tokunboh Odubanjo, Rajiv Swamy, Mohannad Hosny

**Affiliations:** 1 Urology, East and North Hertfordshire National Health Service (NHS) Trust, Stevenage, GBR; 2 Pathology, East and North Hertfordshire National Health Service (NHS) Trust, Stevenage, GBR

**Keywords:** testicular mass, giant liposarcoma, paratesticular liposarcoma, paratesticular tumors, dedifferentiated liposarcoma

## Abstract

Giant paratesticular liposarcoma (PLS) is an uncommon tumour, often misdiagnosed pre-operatively, which presents as a painless scrotal mass. Early detection and prompt surgical management provide the best outcome. We present an 87-year-old patient with gradually enlarging, painless left scrotal swelling. Ultrasound on initial presentation suggested a benign hernia, resulting in an 11-month treatment delay. Computed tomography (CT) thereafter showed paratesticular scrotal mass measuring 14 x 8 x 7cm. Radical inguinal orchidectomy with high ligation of the spermatic cord was performed. Histopathology and cytogenetics confirmed PLS with both de-differentiated and well-differentiated features involving the spermatic cord margin. The patient had rapid progression to fatal lung metastasis within three months of surgery. Our case highlights that any suspicious fat swelling should be investigated thoroughly and excised promptly if paratesticular liposarcoma is suspected, as delayed management gives poor outcomes.

## Introduction

Urological sarcomas account for roughly 2.7% of all soft tissue sarcomas, with the paratesticular region being the most common site of origin for such tumours [1]. They arise within the scrotum, and do not involve the testes but can include surrounding structures such as the tunica vaginalis, spermatic cord, epididymis, and fascia. Currently, only about 200 cases of paratesticular liposarcoma (PLS) have been reported in the literature, with only a few being giant PLS >10cm [2]. They most frequently present between the fifth and sixth decade and are often misdiagnosed as scrotal hernia at initial presentation. Mainstay treatment involves surgery in the form of inguinal orchidectomy and high ligation of the spermatic cord. There is no consensus on the adjuvant treatment of such aggressive malignancies due to their rarity. We report on a rare, aggressive case of giant, high-grade de-differentiated PLS complicated by rapid progression to fatal lung metastasis within three months of surgery.

## Case presentation

An 87-year-old patient presented to his General Practitioner (GP) with a small left testicular lump 11 months ago. Ultrasound scan (USS) in September 2020 showed a small left scrotal lump with an area of echogenic tissue traceable cranially into the groin, suspicious for a fat containing hernia. The patient was managed conservatively by the GP as the 'hernia' was small and not causing any pain. Eleven months later, the patient was seen by the general surgeons in the emergency department for suspected incarcerated left inguinal hernia. The patient gave a history of gradually enlarging, painless left testicular swelling with no history of testicular trauma, lower urinary tract symptoms or urethral discharge. 

**Figure 1:**
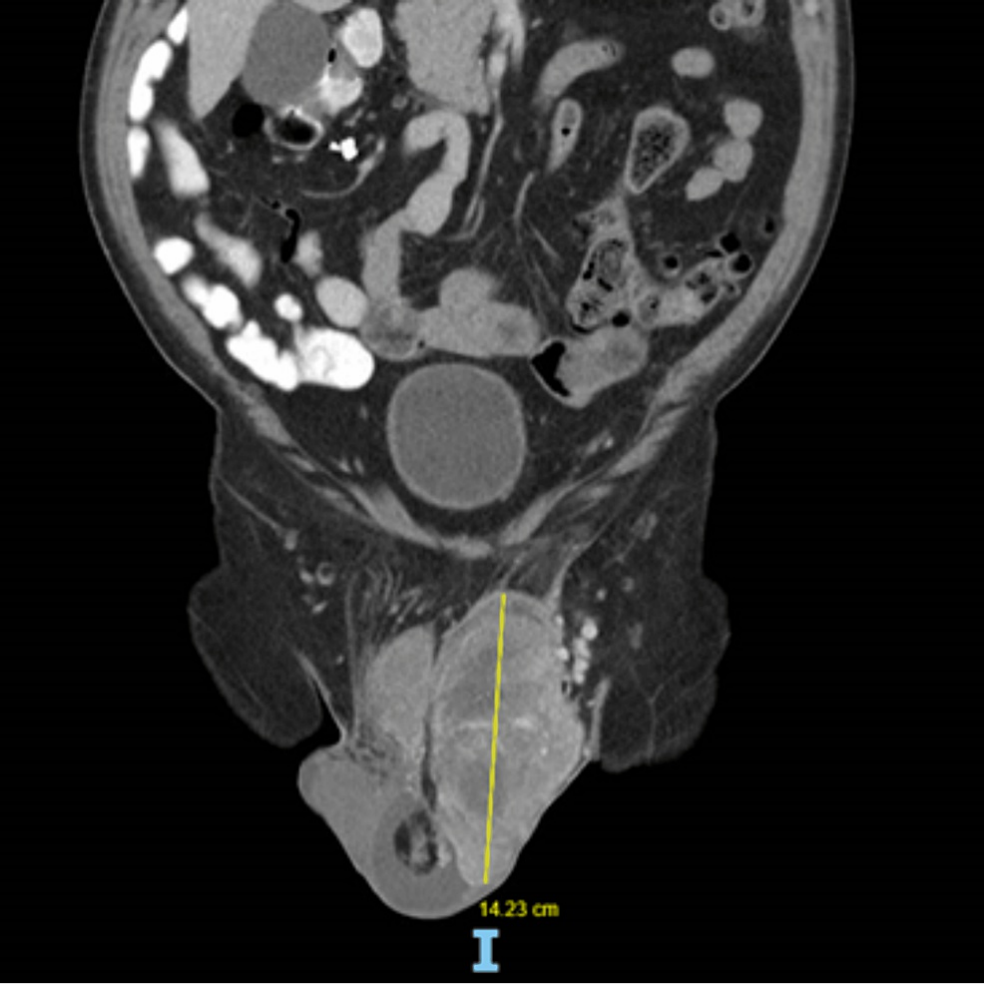
CT abdominal-pelvis scan The image is showing left paratesticular liposarcoma measuring ~14cm.

On examination, there was a hard lump arising from the upper pole of the left testis, which occupied the whole left hemiscrotal area and displaced the right testis. The upper border of the mass was palpable in the left inguinal area. The overlying skin showed signs of hypervascularity. A repeat doppler USS scan in August 2021 showed a large mass measuring 13 x 9 x 10cm with mixed echogenicity and vascularity attached to the left testes. Based on this finding, the patient was referred to the Urology team for management. The patient’s case was discussed in the radiology-urology multidisciplinary team (MDT). Tumour markers (lactate dehydrogenase, alpha-fetoprotein, and human chorionic gonadotropin) for testicular cancer were all negative. Due to diagnostic uncertainty, an abdominopelvic CT was performed which showed a left paratesticular scrotal mass measuring 14 x 8 x 7cm (Figure 1), pushing the left testicle caudally. A staging CT-thorax showed no evidence of thoracic metastatic disease.

**Figure 2:**
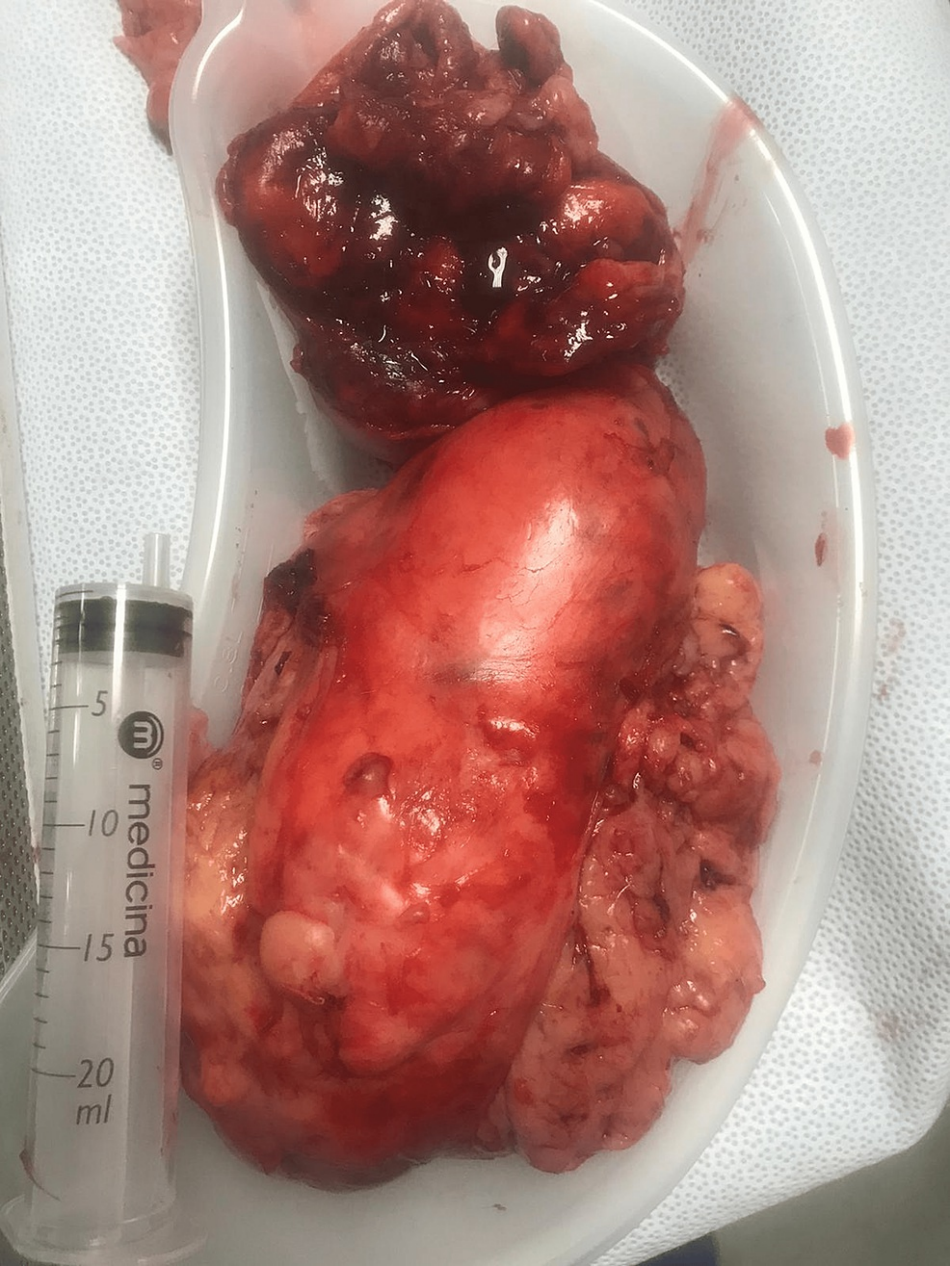
Giant fleshy lobulated paratesticular liposarcoma

The patient proceeded to have radical left inguinal orchidectomy and high ligation of the spermatic cord (Figure 2). Intra-operatively, an inguinal incision was made and we were able to identify the spermatic cord, which was adherent to the surrounding structures. After high ligation of the spermatic cord, the large specimen was delivered. The specimen included the whole cord, the tunica vaginalis surrounding the left testis, and the tumour inside. Macroscopically, the tumour appeared as a fleshy lobulated mass with focal areas of haemorrhage and necrosis, measuring 17 x 15 x 8cm.

**Figure 3:**
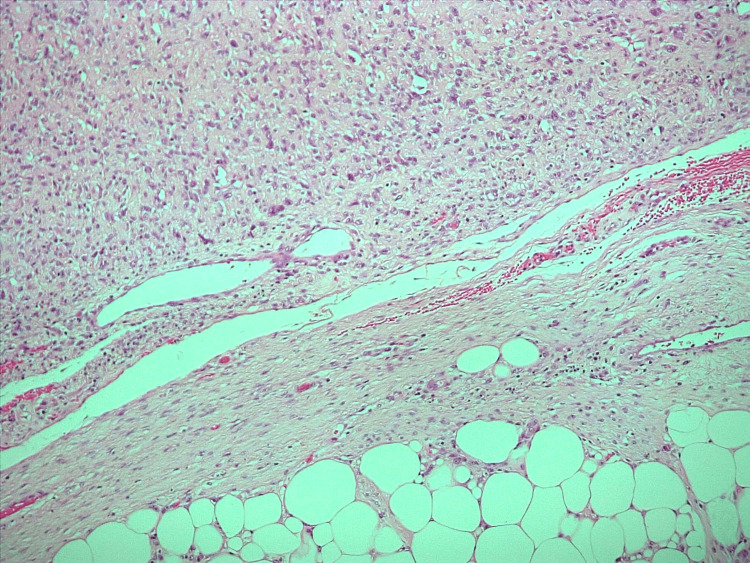
Immunohistochemistry image H&E 10X The image shows a transition from well-differentiated to de-differentiated liposarcoma

The tumour showed a paratesticular topography with no actual involvement of the testicular parenchyma. Microscopically, the tumour showed sheets of spindled to epithelioid cells arranged in fascicles, focal areas of storiform and herringbone patterns, with a striking lipomatous component. Mitotic index was 20/10HPF (high power field of the microscope) cells and markedly pleomorphic bizarre nuclei were present. Immunohistochemistry showed that the tumour cells were diffusely positive for desmin and vimentin but negative for MyoD1 and myogenin. The morphology and immunohistochemistry supported a liposarcoma with areas of de-differentiation (Figure 3). *MDM2* gene amplification demonstrated by fluorescence in situ hybridization confirmed the diagnosis of liposarcoma.

**Figure 4:**
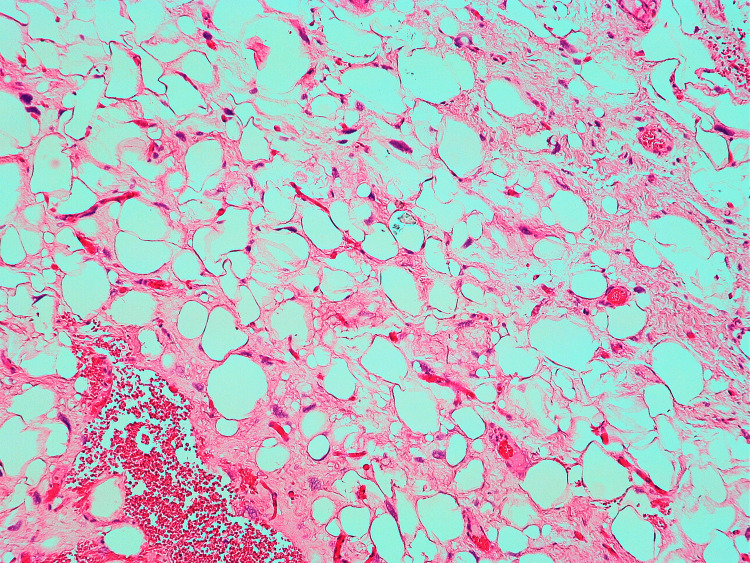
Spermatic cord margin involved by well-differentiated liposarcoma H&E 10X

The tumour involved the mesothelial surface of the tunica vaginalis and well-differentiated liposarcoma (WDL) was seen in the spermatic cord (Figure 4) right up to the resection margin (R1). There was no involvement of the testis, rete testis or epididymis seen microscopically. The final pathological staging was pT4 (tumour size>15cm).

Due to the rarity and stage of tumour, this was referred to a specialised sarcoma unit for MDT discussion. However, before an outcome was reached, the patient was admitted with left hemi-scrotal swelling and new cutaneous lesions around site of previous orchidectomy, five weeks post-operation. CT chest-abdomen-pelvis showed metastatic lung deposits and local disease progression, with cutaneous recurrences found around left inguinal and hemi-scrotal region. The patient was seen by the oncology consultant whilst an inpatient. Unfortunately, chemotherapy was not a viable option in view of the patient’s frailty. After discussion with the patient and his family, a decision was made to transfer the patient to a hospice for palliative management. The patient died shortly after, with time of diagnosis to death being 101 days.

## Discussion

Liposarcoma is a soft tissue malignancy derived from mesodermal tissue embryologically. It is the most common histological type (46%), followed by leiomyosarcoma (20%), histiocytoma (13%), and rhabdomyosarcoma (9%) [3]. Tumour size approaching >10cm is defined as ‘giant’, with only a few cases being described in the literature [2]. In the 2020 WHO classification of soft tissue tumours there are five reported types of malignant adipocytic tumours: well-differentiated (WDL), de-differentiated (DDL), myxoid, pleomorphic, and myxoid pleomorphic [4]. WDL and DDL are the most common liposarcomas in the paratesticular region, constituting 40-45% and 10% of cases respectively [5]. WDL and DDL represent a histological and behavioural spectrum of a single disease entity [6,7]. Whilst some DDL can arise de novo, some occur as a recurrence of WDL [5]. WDL and DDL are cytogenetically related, sharing the same basic genetic abnormality of amplified sequences originating from the long arm of chromosome 12 [6,7]. They have a similar clinical presentation but differ in their clinical course. WDL can recur, does not metastasize but can dedifferentiate to DDL. DDL exhibits aggressive clinical behaviour with the potential to metastasize and is associated with a poor prognosis. Local recurrence and metastatic rate for DDL have been reported as 40% and 15-30% respectively [5]. Therefore, histopathological diagnosis is important in allowing clinical course prediction and thereby follow-up.

Excision specimens of any potential liposarcoma should be carefully observed and sampled, because tumour diagnosis and grading may depend on features that are focal within the specimen. Resections from retroperitoneal/paratesticular sites, in particular, should be thoroughly sampled and assessed to avoid misdiagnosis of DDL as malignant fibrous histiocytomas (MFH) or undifferentiated pleomorphic sarcomas (UPS) which they can morphologically mimic. When compared with UPS or leiomyosarcomas, DDL have a lower tendency toward local recurrence and metastasis [8]. Most of the DDL samples show areas of WDL allowing for its accurate diagnosis as seen in our case. Furthermore, with a growth rate of 1.1cm/month, our case fits with the clinical course of DDL. 

Clinical diagnosis of PLS is difficult as it presents similarly to inguinoscrotal hernia and lipoma in the form of painless left inguinoscrotal mass. Therefore, radiological imaging is required to guide diagnosis. USS can help identify solid, hyperechoic and heterogenous lesions but cannot accurately distinguish lipoma from a small liposarcoma [3]. In our case, lack of clinical suspicion, misdiagnosis of hernia on USS and delayed surgical intervention attributed to the poor patient outcome. MRI remains the gold standard investigation for soft tissue tumours as it helps characterise and delineate the degree of local tumour extension and thereby staging [3]. MRI was not performed in our case pre-operatively as PLS was not suspected initially.

There is no universal consensus on the management of giant PLS due to its rarity. Radical orchidectomy, wide local excision and high ligation of the spermatic cord provide the best outcome [2]. Three-year local-recurrence-free survival has been reported to be 100% for negative margins and 29% for positive margins [2]. Risk factors for local recurrence include de-differentiated subtype, high-grade disease, and tumour size >5cm. Repeat wide local excision is often required to manage local recurrence [9]. The value of adjuvant therapy remains debatable. Combined surgical treatment and adjuvant radiotherapy have been recommended for patients with a high risk of locoregional recurrence or positive margins in a cohort study [9]. The value of chemotherapy is to be determined, as no large clinical trials on PLS have been conducted yet.

Our case highlights that DDL is associated with a high rate of local recurrence and metastasis, in comparison to WDL which is of better prognosis. Therefore, any suspicious fat swelling should be investigated appropriately, even if asymptomatic, and excised promptly to prevent progression into DDL.

## Conclusions

Giant PLS is a rare entity and is often misdiagnosed resulting in treatment delay. The gold standard imaging for PLS is MRI, with definitive diagnosis provided by histopathological studies. Radical orchidectomy and high ligation of spermatic cord should be performed as soon as possible if PLS is clinically suspected, to prevent progression of WDL to DDL. Regular follow-up is needed in cases of DDL in view of its metastatic potential. Chemoradiotherapy can be offered on an individual basis, although the evidence in literature remains limited.
